# Inequities in the delivery of mental health care: a grounded theory study of the policy context of primary care

**DOI:** 10.1186/s12939-021-01492-5

**Published:** 2021-06-19

**Authors:** Rachelle Ashcroft, Matthew Menear, Jose Silveira, Simone Dahrouge, Monica Emode, Jocelyn Booton, Kwame McKenzie

**Affiliations:** 1 Factor-Inwentash Faculty of Social Work, University of Toronto, 246 Bloor Street West, Toronto, Ontario M5S 1V4 Canada; 2 Department of Family Medicine and Emergency Medicine, Faculty of Medicine, Université Laval, Quebec, Quebec Canada; 3 Department of Psychiatry, Faculty of Medicine, University of Toronto, Toronto, Ontario Canada; 4 Department of Family Medicine, Faculty of Medicine, University of Ottawa, Ottawa, Ontario Canada; 5 School of Population and Public Health, University of British Columbia, Vancouver, Canada; 6 Wellesley Institute, Toronto, Ontario Canada

**Keywords:** Primary care, Mental health care, Policy, Equity, Qualitative

## Abstract

**Background:**

Strengthening capacity for mental health in primary care improves health outcomes by providing timely access to coordinated and integrated mental health care. The successful integration of mental health in primary care is highly dependent on the foundation of the surrounding policy context. In Ontario, Canada, policy reforms in the early 2000’s led to the implementation of a new interprofessional team-model of primary care called Family Health Teams. It is unclear the extent to which the policy context in Ontario influenced the integration of mental health care in Family Health Teams emerging from this period of policy reform. The research question guiding this study was: what were key features of Ontario’s policy context that influenced FHTs capacity to provide mental health services for mood and anxiety disorders?

**Methods:**

A qualitative study informed by constructivist grounded theory. Individual interviews were conducted with executive directors, family physicians, nurse practitioners, nurses, and the range of professionals who provide mental health services in interprofessional primary care teams; community mental health providers; and provincial policy and decision makers. We used an inductive approach to data analysis. The electronic data management programme NVivo11 helped organise the data analysis process.

**Results:**

We conducted 96 interviews with 82 participants. With respect to the contextual factors considered to be important features of Ontario’s policy context that influenced primary care teams’ capacity to provide mental health services, we identified four key themes: i) lack of strategic direction for mental health, ii) inadequate resourcing for mental health care, iii) rivalry and envy, and, iv) variations across primary care models.

**Conclusions:**

As the first point of contact for individuals experiencing mental health difficulties, primary care plays an important role in addressing population mental health care needs. In Ontario, the successful integration of mental health in primary care has been hindered by the lack of strategic direction, and inconsistent resourcing for mental health care. Achieving health equity may be stunted by the structural variations for mental health care across Family Health Teams and across primary care models in Ontario.

## Background

Efforts to expand team-based models of primary care have potential to increase primary care’s capacity for mental health care by bringing family physicians and nurse practitioners together with mental health providers such as social workers, mental health counsellors, psychologists, psychiatrists, and others [1, 2]. Yet it is unclear the extent to which primary care teams that emerged from a period of policy reform were prepared to integrate mental health care during a period of transformational change.

Organizational theory highlights that delivery system changes – such as integrating mental health in primary care – occurs within a broader context [3, 4]. The broader context involves a constellation of relationships and interactions between the organization and external policy actors; social and economic conditions; and the organizational, institutional, legislative, and policy structures that enable and constrain allocation of resources [3–9]. Appropriate policy and legislative frameworks are necessary for achieving successful integration of mental health services in primary care [10–12]. The policy context, however, is a neglected focus of primary care research [3]. Little remains known about how the policy context encouraged or deterred the integration of mental health during a period of primary care reform.

### Primary care reforms

Policy reforms in the United States and Canada taking place over the last two decades have aimed to improve access and strengthen prevention and management of chronic diseases [1, 5, 13–15]. In the 2000’s, there was growing consensus about the benefits of coordinated care and interprofessional primary care teams [16–18]. Family Health Teams (FHTs) are one example of an interprofessional team-based model of primary care that emerged from a period of policy reform in Ontario – Canada’s most populous province [14, 16, 18]. The first wave of FHTs were launched in 2005, with 186 FHTs currently caring for approximately 3 million Ontarians [18–20]. The operationalization of FHTs occurred within one of five waves, or time-periods, between the years 2005–2012 [18, 21]. FHTs intended to improve access and quality to comprehensive care by increasing the range and types of available services through the implementation of teams comprised of diverse interprofessional healthcare providers (IHPs) [14, 22]. Facilitating the expansion of team-based practices, primary care reform in Ontario implemented new funding for IHPs, shifted physician remuneration from fee-for-service to a capitation-based system, and introduced new financial incentives [14, 16, 22]. Robust policy reforms reshaped primary care in Ontario and elsewhere [16, 23]. New initiatives are currently underway to reorganize the health system with aims to provide a more coordinated continuum of care across sectors, including mental health [24–26]. It is unclear, however, the extent to which policy reforms encouraged and supported primary care’s capacity for mental health care.

### Mental health equity in primary care

There is an overwhelming need to strengthen access to mental health care across the globe [10, 27, 28]. The prevalence of patients with depression and anxiety in primary care is high [12, 29, 30] yet a treatment gap for mental health continues to persist [31–33]. Addressing such mental health disparities requires improved access to preventative and treatment-focused mental health care [34, 35]. Primary care is optimal for early identification, treatment, management, education and counseling, relapse prevention, and coordination of common mental disorders - like depression and anxiety - because it is person-centred, comprehensive and community-based [1, 2, 10, 36–38]. Patients build long-term relationships with their primary care providers, allowing these professionals to develop unique insights that assist with diagnosis, treatment, and follow-up [38]. Strengthening primary care’s capacity is one of the most effective approaches for meeting population need for mental health care and health equity [2, 39–43].

Strengthening primary care’s capacity for mental health care improves health equity outcomes by providing timely access to coordinated and integrated mental health care [10, 43, 44]. Health equity refers to the absence of preventable and unjust disparities between different populations [45]. Horizontal equity refers to the principle whereby individuals who have the same level of need should then receive the same level of services [45, 46]. Whereas, vertical equity refers to the principle that individuals with greater need should receive higher priority and attention for health care [45, 46]. Policy reforms in the early 2000’s were intended to improve some of the shortfalls that existed by improving access to primary care teams [47]. To this extent, policy reforms in Ontario enhanced horizontal equity as it relates to first-contact access. It is unclear, however, the extent that policy reforms addressed the mental health treatment gap and facilitated integration of mental health care in primary care teams. Without this level of understanding, unintended consequences and obstacles can arise for future policy reforms [16].

### Rationale for study

There is a large body of literature focusing on the internal organizational contexts of primary care practices; yet, there is a considerable gap about the policy context [3, 48]. Early research suggested that the lack of attention to mental health care in policy might have deterred widespread integration of mental health in primary care during a period of health system transformation [49, 50]. Primary care research that takes into account the wider policy context can help to move to a more robust explanation of what works for whom and under what circumstances [3, 51, 52]. Our study aimed to provide a more robust understanding of the policy context that shaped one primary care model’s capacity for mental health delivery in Ontario. This study was part of a larger qualitative study investigating the incentives that influence the quality of mental health care in Ontario’s FHTs [53]. To proceed with our investigation of incentives, we needed to understand the policy context within which primary care teams in Ontario were situated [54]. This paper presents findings related to one question of our study: What were key features of Ontario’s policy context that influenced FHTs capacity to provide mental health services for mood and anxiety disorders?

## Methods

### Study design

We used constructivist grounded theory to guide our study [55, 56]. The inductive method of constructivist grounded theory is well suited for pursuing new insights from multiple viewpoints without imposing pre-existing constructs [55, 56]. Our research team was comprised of members representing different clinical or disciplinary backgrounds spanning: social work, psychiatry, mental health research, epidemiology, and primary care health services delivery research. Two members of the team had experience as advisors to provincial policy and decision-makers. Sensitizing concepts helped to inform the research process by providing a starting point [55–57]. We derived sensitizing concepts from our previous research [18, 49, 58].

### Study sample and recruitment

The 186 MOHLTC-funded FHT organizations in Ontario, Canada represent the sampling frame for this study. Providers and administrators (i.e. executive directors, program managers) from any of the 186 FHT organizations were eligible to participate and identified using the list of FHTs made publically available by the MOHLTC [59]. Eligible FHT providers and administrators included executive directors, family physicians, nurse practitioners, nurses, and the range of professionals who were involved in the delivery of mental health services within FHTs. Provincial policy and decision-makers, and key community stakeholders were also eligible to participate in this study.

Consistent with constructivist grounded theory, we used both initial and theoretical sampling [53, 55, 56]. At the initial sampling phase, we strived to include participants representing diversity in the FHTs included in our sample with respect to rural and urban location, team size, and type of provider composition on the team. For team composition, we strived to recruit participants from FHTs who had both higher and lower mental health services. For the purpose of this study, we defined those FHTs with two or more different types of mental health providers as having higher mental health service capacity, and those with less than two different types of mental health providers as having lower mental health service capacity. This distinction was only determined to help with sampling, and was not to suggest that FHTs in either of those categories provided higher or lower quality of mental health care to their patients. The aim of this distinction was to achieve a diverse sample reflective of the diversity that exists across FHTs in terms of size and provider composition.

We recruited potential participants by emailing invitational letters to executive directors at FHTs, provincial policy and decision makers, and some key community stakeholders. Those interested in participating in the study responded by contacting the first author (RA) and/or the research coordinator by email or telephone. All interviews were conducted in-person and scheduled at a time and location most convenient to participants.

### Data collection

Two authors conducted all interviews (RA/JB). Interviews ranged in length from 27 min to 90 min, with the majority of interviews being approximately 60 min. Interviews were audio-recorded with participants’ consent, and transcribed verbatim immediately following the interview. Following the completion of each interview, memo-writing occurred by the person who conducted the interview [55].

Data collection occurred in two phases. The focus of phase one of data collection was descriptive (initial sampling), whereas interviews conducted in phase two of data collection were intended to provide deeper understanding and explanation from what we learned in the earlier interviews (theoretical sampling). Because our study aimed to understand the relationships across multiple concepts [60], saturation occurred at 96 interviews. We conducted interviews between April 2016 and October 2018.

### Data analysis

Data collection and data analysis occurred in parallel [53, 61]. Analysis began immediately following transcription of each interview. We used the grounded theory approach of data analysis including initial, focused, and axial coding [53, 55]. Sensitising concepts identified in our scoping review and pilot data helped the initial process of coding data [58, 59]. The first analysis step involved line-by-line open coding of interviews [55, 61]. Focused coding was the second step of coding which entailed comparison of codes, and data were broken into components of properties and labelled [55, 61]. Axial coding refers to the phase where we identified relationships between the constructed categories [55].

We conducted data analysis using a collaborative team coding process, and held monthly meetings with the data analysis sub-committee (RA/MM/JB/ME). We developed a list of coded concepts to help characterize the influential contextual factors, through iterative discussion of the transcripts by the data analysis sub-committee, as well as extensive discussion with all members of the research team at monthly meetings. Throughout this process, we used memo-writing to inform our analyses [55]. The electronic data management programme NVivo11 helped organise the data analysis process. We presented findings to our community advisory committee - comprised of experts in mental health and primary care - at three different time-points as a type of member-checking strategy [62]. We kept an audit trail throughout the duration of the study, systematically recording the data collection and data analysis processes [63].

### Ethical considerations

Ethics approval was received Research Ethics Board Approval from the University of Waterloo (#20973), University of Toronto (#33175), the Centre for Addiction and Mental Health (CAMH) (#140/2015), Bruyère Continuing Care (#M16–16-001), St. Joseph’s Health Centre/Unity Health Toronto (#15–830), and Université Laval (#2016–2877). This study complied with the principles of voluntary and informed consent. Participants were provided with information about the study (aims and objectives), and invited to participate. Prior to commencement of each in-person interview, we reviewed and obtained informed consent. Each participant received a copy of the information and consent form. This study adhered to confidentiality of data by removing participant identifiers from transcripts, data stored in a secure location at the University of Toronto and accessible to researchers only. Participants were provided with a copy of their de-identified transcript by email to ensure that transcripts were de-identified to the participant’s satisfaction.

## Results

We conducted a total of 96 interviews with 82 participants. We interviewed 14 participants twice – once in phase one of data collection, and once in phase two. Of the 82 participants, 65 were FHT providers and administrators, 9 were policy informants, and 8 were key community stakeholders. Table 1 provides an overview of the participant roles.

**Table 1:** Professional Roles of Participants (*N* = 82)

Participant Roles (***N*** = 82)	
**Family Health Team Participants: Providers and Administrators**	***n*** **= 65**
Social Work^a^	14
Family Physician	11
Executive Director	10
Mental Health Counsellor	9
Psychiatrist	7
System Navigator	3
Nurse	2
Nurse Practitioner	2
Occupational Therapist	2
Program Manager^a^	2
Psychologist	2
Outreach Worker	1
Pharmacist	1
**Policy Informants**	***n*** **= 9**
**Key Community Stakeholders**	***n*** **= 8**

^a^One participant held two part-time roles in the same FHT

Participants provided insights of the contextual factors they considered important features of Ontario’s policy context that influenced FHTs capacity to provide mental health services for mood and anxiety disorders: We identified four themes in the data: i) lack of strategic direction for mental health, ii) inequitable resourcing for mental health care, iii) FHT rivalry and envy, and, iv) variations across primary care models.

### Lack of strategic direction for mental health

Participants emphasized the need to make mental health care a provincial priority in order to provide a strategic vision for mental health care across Ontario FHTs. As one policy informant stated, “*At the provincial level it is about strategic direction, it is about broad priorities, it is about charting the path forward*” (P46). Participants explained that there was an explicit provincial priority to improve access, which facilitated the establishment of a common goal across FHTs. One FHT administrator explained: “*Access to [a] regular primary care provider, that was a big priority … .We have done an excellent job of addressing that across the FHTs*” (P2). Participants considered the role of provincial priorities important for informing coordinated planning, helping identify shared-goals, and providing direction for provincial funding decisions. Many of our participants expressed concern that policy makers did not understand primary care. An executive director emphasized that there was a lack of “*a shared [provincial] understanding of what is the role of primary care*” (P64). A policy informant elaborated and reported that there was a lack of “ … *clarity around the model and what it should accomplish with a mental health lens*” (P57). According to our participants, there was no explicit direction provided to new FHTs related to mental health care. A policy informant explained:

*When we started with the Family Health Team initiative, we described the model, we had a sort of competitive call for applications process to determine who was going to be eligible to get the funding to support that model and kind of said, ‘here’s a contract, here’s some guidance documents, go and create an organization. Go and design programs’ … I think there’s a solid lesson learned there* (P46).

An executive director agreed, “*Family Health Teams kind of ran with what they had and there wasn’t a lot of thought given to how do we allocate resources*” (P34). FHTs had different approaches in developing teams as explained by an executive director: “*I think a lot of [FHTs] … have kind of gone for the cheapest services a lot of the time rather than the most needed services*” (P74). The lack of strategic direction and guidance meant that FHTs varied in their abilities to operationalize. “*Some Family Health Teams really grabbed onto that, and have done very well. Others have struggled, and probably needed more support than what was provided*” (P46).

Most participants expressed concern about the absence of provincial priorities for primary mental health care. One participant stated, “*I’m actually on the committee that looks at what the Ministry is targeting, and mental health isn’t there at all … .if it’s not prioritized at all, it’s not even on the table*” (P63). All participants reported that there was not enough attention given to mental health in primary care by provincial policy and decision-makers. One participant who was a FHT provider stated, “*there hasn’t been a concerted effort into managing [mental health] on a provincial level*” (P10). Another participant who was a psychiatrist explained, “*I think there is really a lot of room in terms of [MOH], bigger picture type things to, to try to use the evidence to make a great mental healthcare system*” (P43). A family physician agreed, “*We talk a big talk about mental health but like nothing is actually executed*” (P25).

### Inequitable resourcing for mental health care

Participants overwhelmingly spoke about inequitable funding across FHTs, and the implications this had on long-term mental health resourcing in FHTs. Throughout the interviews, participants spoke about the difference of resources that occurred across the five waves, or time-periods, between the years 2005–2012. “*Family Health Teams were first announced in 2005, money started to flow in late 2006, the first Family Health, the first batch of fifty Family Health Teams came up at that point in time*” (P44). Participants explained that the amount of funding made available lessened over time, which meant there was more funding for FHTs that became operational in the earlier waves than for FHTs that became operational in later waves. One participant noted: “*I … watched the progression of very heavily funded larger teams, and then it kind of trickled down as they were getting to their promise of 200 Family Health Teams, the resources were a lot less than kind of they were before*” (P44). This policy informant explained the rationale for the different funding levels: “*Funding decisions were made by the time they got to waves four and five, the economy had turned in a different direction, money was really tight, and they did not receive the same kind of funding as wave one*” (P44). In addition, participants noted that FHTs that became operational in later-waves had less *base* funding:

*The numbers of positions that were approved got smaller and smaller with the waves, so that the, just the person power capacity [for mental health care] when it comes to the IHP side would be less so in wave five … They’re now stuck with it … as base funding and one time funding. As soon as something is approved for the base, it continues on. So if you had all of these things in your base and you were wave one, its carrying on. If you were in wave five and you are asking for and you’re pleading for additional funding, there’s no more money. So you have to continue what was approved in your base, which was a smaller base to begin with … so there was … different waves, different amounts.* (P44)

The variation in funding levels over time helps explain some of the human resources differences that exist across FHTs in terms of the variations in provider numbers and types. One participant who was a provider from a wave five FHT stated, “*We were one of the last Family Health Teams to be added in the province, so we have maybe not the most robust staffing complement*” (P61). The implications was that FHTs in the later waves had less resourcing to hire mental health providers. An executive director explains: “*Waves 2 and waves 3 … didn’t have access to the same resources that we did … So for example, the [Name of Nearby FHT] didn’t put the same emphasis on mental health … they just don’t have the same complement of staff that we do*” (P64). Participants explained that one of the difficulties was that these inequities across FHTs were not always recognized. A policy informant explained:

*There is a predisposed bias towards FHTs … the assumption that all FHTs have equitable, similar structures, similar access to resources. No one ever thinks there’s inequity spread across FHTs … That’s the first misperception that has to be kind of corrected. There is inequity across the province* (P81).

Despite FHTs in later waves not having the same level of resourcing for mental health professionals as earlier FHTs, participants explained that the demand for mental health care remained high for all FHTs. A FHT provider noted, “*If you’re like third or fourth wave, you’re not going to get funding for more but the need is still there*” (P56). Another participant who was a family physician agreed, “*There’s no way that primary care practitioners are not doing the bulk of mental health delivery. We are doing the bulk of mental health delivery*” (P6). Yet mental health care in FHTs remained underfunded particularly for the FHTs that emerged in the later waves. According to participants, funding for mental health providers has not aligned with service demands. An executive director stated, “*It all comes down to money … money for more mental health resources, like human resources*” (P28). Another executive director noted that there’s “*very little funding to hire mental health professionals*” (P50). All participants, however, expressed concerns about mental health care having been underfunded. An executive director reported, “They talked about diabetes and they talked about hypertension, they talked about all kinds of other things, but mental health … has been underfunded in most jurisdictions, stigmatized, and therefore under resourced” (P74).

### FHT rivalry and envy

No two FHTs are alike. Participants explained that there are a wide-range of differences that existed across FHTs. For example, a FHT administrator stated: “*There’s a lot of … differences in those teams in the make-up of their teams, and how long they’ve been established … if they are academic or community or physician led. Or, like all different things kind of change the characteristic of that team*” (P11). Despite the model’s aim to improve collaboration, many participants explained that the variations in funding and differences across FHTs nurtured a context, at least in some cases, of FHT envy and rivalries. “*The other Family Health Team in this town … is a physician led board, this one is a community led board. We do our own thing and try our best to give the best care we possibly can. There’s an ongoing dislike from that team*” (P24). Many participants spoke at length about other FHTs who they perceived to have more mental health resourcing than they did. For example, one executive director stated, “*If you look at the team to our south. They have a psychiatrist on staff*” (P50). Participants in rural communities in particular spoke about the difficulties they encountered because of the ensuing rivalry. One participant explained, “*Rivalry maybe that’s the word I don’t know … I do think that there’s some sort of, us versus them scenario going on in this small town*” (P24).

### Variations across primary care models

Many participants expressed concern that the variations of primary care models across Ontario meant that only patients who were rostered to a team-based model – like the FHTs - had access to the interprofessional providers and mental health resources in these teams. A policy informant stated, “*The province having only twenty-five percent of the population able to access doesn’t make a heck of a lot of sense*” (P44). Another policy informant agreed, “*The haves and the have nots, so those, those physicians and those patients that have access to Ministry funded allied health professionals, versus those that don’t*” (P46). Most participants explained that not all family physicians in Ontario had the same opportunities of direct and indirect support of the mental health providers to the extent that physicians in FHTs did. According to a psychiatrist:

*A lot of resources tend to get focused within Family Health Teams versus the solo providers in a community because they don’t have access to … the indirect care time and the opportunity to have the improved access that having a psychiatrist implanted in the team allows … .I think that’s a big issue in Ontario … there’re lots of primary care providers out there who aren’t part of Family Health Teams*” (P43).

Participants explained that the different levels of mental health resourcing across the different primary care models in Ontario meant that patients across Ontario did not have access to same amount and types of mental health services. A FHT provider stated, “*Those 35,000 patients, they have access to eight sessions of free mental health treatment, and people who aren’t in a Family Health Team don’t*” (P65). Another participant who was a family physician reported that the discrepancy of resourcing was in part dependent on the primary care physicians’ choice to opt into the team-based model. “*If your doctor in southern Ontario who chooses not to practice in one of these different groups then your patients do lose access to those services, those allied services that would be provided in in the group*” (P36). Several participants raised concerns that the variations across models was a disservice for patients. A family physician reported, “*I think this whole ‘physicians who have these resources and physicians who don’t’ is completely unethical from a patient perspective*” (P75). Participants emphasized the need to ensure that all patients’ across Ontario have equitable access to mental health services when needed. A family physician explained, “*I actually think we need to decide on what the level of access should be and make sure we have it across the province, regardless of who your physician is*” (P75). A policy informant agreed:

*The big issue is not all family doctors and their patients have equal access to resource to meet patient needs. FHTs are always thought of as being the crème de la crème, and they have all of this, everything from psychiatrists contracted on a session basis, to the social worker down the hall … So they have a luxury of available resources that a lot of other family doctors don’t have for their patients. Who’s to say that physician’s patients aren’t equally deserving of options that a FHT patient has access to!*” (P81)

## Discussion

Our study provides qualitative insights into the key features of Ontario’s policy context that influenced FHTs capacity to provide mental health services for mood and anxiety disorders. Participants in our study spoke passionately about the need for greater guidance from provincial policy for mental health care so that they could better respond to the needs of their patients and communities. By examining the policy context, our study responds to Thomas’ [3] urgent call for primary care research that focuses on contextual factors. What was surprising in our study was the extent of the variations that existed for mental health care across primary care. These variations may not be evident to clinicians without the broad contextual view that a study like ours provides. Clinicians struggling to meet the mental health care demands of their patients’ may not grasp the extent to which contextual factors informs their clinical capacity.

Findings from our study provides a foundational framework to understanding deficiencies that lead to a lack of desired outcomes and unintended consequences. Our study identifies key features to inform policies that intend to support and build capacity for mental health in primary care. Participants pointed to the complexities of the policy context that enabled and/or constrained the capacity of primary care teams to respond to patients’ mental health care needs. Such features included prevailing values and attitudes [3, 9, 51]; economic and political climates [7, 9]; policy structures [48, 51, 64, 65]; and mechanisms used to implement policy changes [16].

### Strategic direction

Findings from our study highlighted the challenges that exist for primary care teams without adequate strategic planning to guide the integration of mental health services. Strategic planning is a “deliberative, disciplined effort to produce fundamental decisions and actions that shape and guide what an organization (or other entity) is, what it does, and why” [66], p.7. Strategic plans thus help guide activities in a way to meet policy objectives, within budget and time parameters [66]. Furthermore, strategic plans enhance primary care’s capabilities for mental health care by conveying values, objectives, and direction for intended action [1, 67, 68]. Aligned and consistent strategic plans can help drive mental health care quality and efficiency [66]. Despite a more general vision for mental health services to be available and delivered within primary care [69, 70], there was not a clearly articulated detailed strategy and plan for the integration of mental health services. The result is that mental health services has evolved in primary care with little oversight, guidelines, or clear standards; as such, has resulted in broad variations across FHTs.

### Funding

Our study provides a qualitative insight to help understand recent quantitative research on funding for mental health disorders since primary care reforms were implemented [71]. While budget sizes for mental health care can dramatically influence the quantity and quality of available services [68], the lack of continuity in that funding undermines the practices’ ability to deliver care. Financial models were key policy levers used to facilitate primary care reform. Although financial models are a mechanism used to guide desired health system goals [16, 36–38], the lack of consistency in funding for IHPs who do mental health work suggests that there was no explicit outcome goal that policy makers intended to achieve with FHTs in terms of mental health care. The amount of sustainable funding for mental health service delivery can provide opportunities for long-term planning for the integration of mental health services into primary health care [67, 68]. One of the challenges experienced by FHTs in later waves was the lack of sustainable base funding for mental health professionals, thus, limiting later waves capacity for long-term planning for mental health service delivery.

### Quality of care

Although this study did not examine the quality of mental health care in FHTs, it does provide some insights as to why care gaps may continue to exist for mental health in primary care. Quality of care is influenced by the organization of care (structures) and the approach to clinical care delivery (processes) [72, 73]. Despite the high prevalence of depression and anxiety in primary care, few patients are screened, leading to inadequate detection of mental health conditions, and many patients with depression and anxiety do not have access to the necessary services to manage their condition [2, 38, 48–50]. In part, this may be because of some of the contextual features like inadequate organizational resources that create challenges to achieving consistent quality standards across practices [19]. Our findings demonstrated that there was inconsistent structural integration of mental health care in newly emerging FHTs following a period of policy reform. As a result, primary care teams’ and providers’ ability to succeed in meeting mental health care demands vary widely across Ontario due to the lack of necessary supports and resources needed to effectively manage and coordinate mental health care within the organizational practice [12]. Successful outcomes in care, are fundamentally related to the context in which the primary care practice is situated [3, 65]. What emerged from the data was the importance of having a provincial policy context that implements structures and processes that help support primary mental health care.

### Structural inequities for mental health care

One of the concerning findings in our study is that the resource variations across FHTs may be perpetuating an atmosphere of envy, rivalries, and structural inequity. We recognize this as an unintended consequence of Ontario’s primary care policy reform [16, 74]. Unintended consequences can arise at any time during the policy process and may occur because policy is ineffective, counterproductive, or related to other externalities [74]. We believe it is imperative to address the unintended structural inequities arising for mental health in primary care across Ontario. Strengthening the integration of high quality mental health care in primary care settings is a lauded strategy to improve health equity outcomes [10, 44].

When it comes to mental health, however, our study demonstrated that the same level of service was not consistently provided across FHTs, thus did not achieve horizontal health equity in this regard [45]. In some cases, variations of service may be a manifestation of vertical equity [45, 46] whereby some FHTs may be providing greater amounts of mental health care because they service higher needs populations or are located in neighbourhoods with higher needs. Based on our study findings, however, many FHTs appear to be under-resourced for mental health care. As well, there have been some criticism that only focusing on the integration of interdisciplinary teams within physician-based models of primary care deters equity by not attending to the effects of team structures [75]. We agree that greater attention is needed to ensure that the underlying structure of the FHT model is designed to consistently promote high quality mental health care. Ontario has had a challenging history addressing populations’ mental health care needs [76]. We believe that there remains opportunity to improve Ontarians’ mental health care needs. Mental health policies can facilitate strong primary care delivery as well as effective integration of mental health services in primary care [10, 12]. Addressing this important public health problem requires a comprehensive and coordinated response in order to help respond to the demand for mental health services for mood and anxiety disorders [10]. Attending to these structural gaps in the policy context can strengthen primary care’s capacity to respond to patients’ mental health needs, improve patient and population health outcomes, and enhance the overall healthcare system performance [77–80].

### Strengths and limitations

An exceptional strength of our study was the large sample size that included a range of interdisciplinary perspectives represented in team-based primary care, influential policy and decision-makers who have been instrumental in shaping the FHT model, and key community stakeholders invested in mental health and primary care. Our large diverse sample aligned with constructivist grounded theory’s value of multiple perspectives and provided us with a deep understanding of mental health care in FHTs. In addition, conducting the 96 interviews over a 21/2 year period was an asset to the quality of the data we collected because participants became increasingly reflective about the role of contexts in the delivery of mental health care as the provincial election neared in 2018. Lastly, a strength of this study was the range of expertise represented by the research team. We established clear processes for team engagement throughout all phases of the study, which were particularly essential as we progressed through data analysis of such a large sample. We recommend that future researchers undertaking such an endeavor establish and document the processes for team engagement, collaborative approaches to data analysis, and preparation of knowledge translation materials early in the study design.

A limitation is that the focus of this study was on one model of team-based care in Ontario, Canada so findings may not apply to all primary care settings. In addition, the method of recruitment of the participants in this study generates a potential self-selection bias of those with a particular interest in mental health.

## Conclusion

As the first point of contact for individuals experiencing mental health difficulties, primary care plays an important role in addressing population mental health care needs. Our study aimed to provide a more robust understanding of the policy context that shaped one primary care model’s capacity for mental health delivery in Ontario. The four themes identified in the data were: i) lack of strategic direction for mental health, ii) inequitable resourcing for mental health care, iii) FHT rivalry and envy, and, iv) variations across primary care models. In Ontario, the lack of strategic direction and inconsistent resourcing for mental health care appear to have hindered the successful integration of mental health in primary care. Achieving health equity may also be restricted by the structural variations for mental health care across FHTs and across primary care models in Ontario.

## Data Availability

The datasets used and/or analyzed during the current study are available from the corresponding author on reasonable request.
